# Older Adults Show Preserved Equilibrium but Impaired Step Length Control in Motor-Equivalent Stabilization of Gait

**DOI:** 10.1371/journal.pone.0052024

**Published:** 2012-12-18

**Authors:** Julius Verrel, Martin Lövdén, Ulman Lindenberger

**Affiliations:** 1 Max Planck Institute for Human Development, Center for Lifespan Psychology, Berlin, Germany; 2 Department of Psychology, Lund University, Lund, Sweden; 3 Aging Research Center, Karolinska Institutet, Stockholm, Sweden; National Cancer Institute, United States of America

## Abstract

Stable walking depends on the coordination of multiple biomechanical degrees of freedom to ensure the dynamic maintenance of whole-body equilibrium as well as continuous forward progression. We investigated adult age-related differences in whole-body coordination underlying stabilization of center of mass (CoM) position and step pattern during locomotion. Sixteen younger (20-30 years) and 16 healthy older men (65–80 years) walked on a motorized treadmill at 80%, 100% and 120% of their self-selected preferred speed. Preferred speeds did not differ between the age groups. Motor-equivalent stabilization of step parameters (step length and width) and CoM position relative to the support (back and front foot) was examined using a generalized covariation analysis. Across age groups, covariation indices were highest for CoM position relative to the front foot, the measure most directly related to body equilibrium. Compared to younger adults, older adults showed lower covariation indices with respect to step length, extending previous findings of age-related differences in motor-equivalent coordination. In contrast, no reliable age differences were found regarding stabilization of step width or any of the CoM parameters. The observed pattern of results may reflect robust prioritization of balance over step pattern regularity, which may be adaptive in the face of age-associated sensorimotor losses and decline of coordinative capacities.

## Introduction

Stable walking depends on the coordination of multiple biomechanical degrees of freedom (DOF) to ensure the dynamic maintenance of whole-body equilibrium as well as continuous forward progression. According to early work on motor control [1] as well as recent theoretical developments [2], [3], successful and efficient motor performance is not reflected in the suppression or elimination of movement variability, but in task-specific coordination across the body, allowing variability in task-equivalent dimensions while minimizing it in task-relevant dimensions. Recently, we analyzed how motor-equivalent coordination among joint angles contributes to the stabilization of CoM position and foot placement during walking in young adults [4]. The present study extends this analysis to normal aging by examining the extent to which differences between younger and older healthy men in motor-equivalent coordination [5]–[7] are present in walking, and whether the pattern of age-related differences reflects functional priorities among performance requirements (e.g., balance versus regularity of the step pattern).

Both the CoM position and foot placement depend on the configuration of many biomechanical DOF (e.g., joint angles) across the body. Due to motor equivalence (the abundance of DOF over relevant task parameters), the same CoM or foot position can be achieved by a large variety of joint configurations [1]. This allows for fluctuations in one joint angle to be compensated by coordinated changes in other joint angles. Relating variability in joint angles to variability in a hypothesized task variable (e.g., step length or CoM position in the case of walking) has been proposed and validated as a way to formally capture the contribution of the central nervous system to the stabilization of this variable [2], [8], [9]. Different analytical approaches have been proposed to this end, such as the uncontrolled manifold (UCM) analysis [8] or the covariation by randomization method [10].

In a recent study of young men, we found that task variables such as step and CoM positions are stabilized by motor-equivalent coordination among the major joints of the body during locomotion [4]. Step-to-step fluctuations in joint angles at the time of heel strike covaried such that task variability was lower than would be predicted from the variability in the individual joint angles. Using the covariation by randomization method [10], we identified control priorities, with the CoM position relative to the front foot showing the strongest covariation, both in the fore-aft and in the lateral direction. At the time of heel strike, body weight leaves the back foot and is transferred to the front foot, making the position of the CoM particularly important for whole-body balance. Thus, the results of that study [4] indicate that the participants exploited motor equivalence to a greater extent for the stabilization of whole-body balance than for the stabilization of step parameters. In addition, by directly analyzing the relationship between CoM position and foot placement, we observed differences between the fore-aft (step length) and lateral (step width) dimension. In the fore-aft dimension, the CoM positions relative to the back and front feet exhibited a negative step-to-step correlation, indicating that they were correlated in a way reducing step length variability, presumably to ensure spatiotemporal regularity of forward progression [11]. In contrast, step-to-step correlations between the two CoM positions in the lateral dimension were consistently high (and positive), in agreement with the view that lateral foot placement compensates for lateral excursions of the CoM to maintain lateral body equilibrium [11]–[14].

Converging evidence suggests that coordination of multi-DOF movements deteriorates with normal aging. In a reaching task involving one or two joints (elbow and shoulder) to experimentally varied amounts, older adults showed reduced smoothness and accuracy with increasing shoulder joint contribution [15]. In a multi-finger force control task during which the total force (sum of individual finger forces) had to be controlled, older participants exhibited not only increased overall variability but also weaker coordination among the fingers stabilizing the total force [5], [6]. In a study on age-related differences in motor-equivalent coordination, we recently analyzed the structure of joint angle variability in manual pointing, using the uncontrolled manifold approach [7]. In that study, older adults showed reduced relative and absolute amounts of goal-equivalent variability, as well as reduced motor-equivalent covariation, indicating reduced use of motor equivalence. A recent paper analyzed adult age-differences in the relationship between step length and step time during treadmill walking [16], finding evidence that older adults made less flexible use of combinations of these two parameters stabilizing walking speed. However, we would like to note that this study analyzed coordination at the level of step parameters rather than at the level of joint angles (or other “elemental” DOF), as discussed above and as planned for the present study.

When multiple functional constraints need to be integrated in a task, such as maintenance of equilibrium (stabilization of CoM position) and forward progression (regularity of the step pattern, e.g. step length control) during walking, reductions in coordinative skill may be reflected in either a general reduction in coordination indices across task variables or, as a more adaptive “solution”, in a selective reduction in coordination only with respect to task variables that are functionally less relevant. To our knowledge, the effects of normal aging on motor-equivalent coordination of the entire body during walking (or any task with many DOF and multiple functional constraints), including associated changes in control priorities, have not been investigated so far.

This study addresses potential age-related differences in the organization of step-to-step variability during treadmill walking in a sample of younger and older healthy men. Coordination was assessed in two ways: First, a covariation index was used to quantify motor-equivalent stabilization of six task-related variables defined at the time of heel strike: step length, step width, and CoM position relative to the back and front foot in the fore-aft and lateral direction. Second, the relationship between CoM and step control was analyzed by computing step-to-step correlations of CoM positions relative to the back and front foot.

We predicted that participants from both age groups would show similar priorities between task variables (assessed by covariation indices) as previously found in a young-only sample [4]. Based on previous work on age-differences in multi-DOF coordination, we also predicted that covariation indices would be lower in older participants for some or all of the task variables.

## Materials and Methods

### Participants

Thirty-two male participants from two adult age groups were recruited for the study, 16 younger men (mean age ± SD: 25.8±2.7 years; body weight: 77.2±8.3 kg; body height: 1.84±0.09 m) and 16 older men (71.4±2.3 years; 74.6±8.2 kg; 1.77±0.05 m). To minimize potential age differences in familiarity with the treadmill, participants from previous studies involving prolonged walking on a treadmill were invited. During the previous studies, they had been exposed to at least two hours of treadmill walking. Participants were screened by telephone interview for conditions that are known to affect balance or gait performance (e.g., Parkinson’s disease, diabetes, gout, severe back pain, impaired balance, cardiovascular problems, artificial hip joint). All participants reported normal or corrected-to-normal vision and hearing. In the lab, the older participants were screened for dementia (MMSE, all scores at least 26/30) [17] and physical disability (SPPB, all scores at least 10/12) [18].

The good physical condition of the elderly participants is further documented by their spontaneous overground speed (measured on a 15m distance) and the self-selected preferred walking speed on the treadmill (determined during the experiment). Though the spontaneously chosen overground velocity was higher in younger participants than in older participants, 5.15±0.45 km/h versus 4.76±0.44 km/h; Welch’s t-test, *t*(30) = –2.48, *p*<0.05, Cohen’s *d* = -0.88, older participants walked at a relatively swift pace. Moreover, no systematic age group difference was found for the preferred speeds on the treadmill [4.49±0.55 km/h versus 4.58±0.44 km/h; *t*(28.6) = -0.50, *p*>0.1].

Written informed consent was obtained prior to the experiment. Each participant received 10 Euros per hour. The Ethics committee of the Max Planck Institute for Human Development, Berlin, approved the study.

### Apparatus and Data Acquisition

Kinematic data were measured using a passive infra-red reflective marker system (VICON, 10 cameras, sampling rate 200 Hz). Reflective markers were placed on relevant anatomical landmarks according to the VICON Plugin-Gait Model (for details, see Verrel et al. 2010), with foot markers attached to running shoes provided by the lab.

Participants walked on a treadmill (Woodway GmbH, Weil am Rhein, Germany), with the walking area (200 x 70 cm) at the level of the surrounding floor. No handrail was present. Participants were secured by a safety harness around the waist. A virtual environment consisting of a straight path was backprojected on a 200 cm x 270 cm screen mounted in front of the treadmill, with visual flow synchronized to the speed of the treadmill with an empirically established flow/speed ratio. During the experiment, the surrounding room was darkened in order to minimize availability of visual references other than the virtual environment.

### Design and Procedure

The experimental session started with a familiarization procedure, during which participants walked at five prescribed speeds (2.4-4.8 km/h), once in ascending and once in descending order (each time 30s). Second, after a short break, participants were asked to find a convenient speed for walking on the treadmill, starting with 75% of the spontaneous overground speed measured before the experiment. Participants had the possibility to adjust the speed (by verbal command), in steps of ±0.3 km/h. To delimit the speed from above and below, each participant was required to walk at the next-lower and next-higher level (±0.3 km/h) before settling on the preferred speed. In ambiguous cases, intermediate speeds (at 0.1 km/h-steps) were presented. This was followed by 60 seconds of walking at the selected speed, with a subsequent possibility to readjust the velocity (not used by any of the participants).

Each participant completed six walking trials of 60 seconds duration each, with two trials at 80%, 100% and 120% of their individual preferred speed. In addition, each participant completed ten walking trials at five prescribed speeds (2.4, 3.0, 3.6, 4.2, 4.8 km/h). The order of these conditions (preferred, prescribed) was counterbalanced across participants. As preferred speeds did not systematically differ between the two age groups (see above) and as the pattern of results was very similar across conditions, only data from the preferred speed condition (80%, 100%, 120%) are reported.

The walking speeds were presented either in ascending-descending or descending-ascending order, counterbalanced between participants and within age groups. Prior to each trial, participants were given time to get used to the current treadmill speed (according to self-report, at least 15 seconds). Then, walking kinematic data were recorded for 60 seconds. Participants were instructed not to talk, turn their head, or make any additional movements during walking. Trials in which such events occurred were repeated immediately (twice during the entire study).

### Data Analysis and Biomechanical Model

All the data analyses were performed using custom-written MATLAB (R2007b, The MathWorks Inc.) routines. The kinematic data were bidirectionally low-pass filtered at 10 Hz with a third-order Butterworth filter. Whole-body postures at the times of heel strike, termed “step postures”, were normalized with respect to the position of the back foot (marker at the first metatarsophalangeal joint, MTPJ). Due to missing markers in some trials, step postures could not be determined for all heel strike events. Therefore, we used only the last 20 complete step postures for each foot in each trial. A biomechanical model was implemented in MATLAB following the specifications of the Vicon PluginGait model [4]. In total, the model comprised 35 joint angles representing the configuration of 15 joints of the upper and lower body. Based on the biomechanical model, the spatial positions and orientations of all the segments (lower limbs, upper limbs, pelvis, trunk and head) can be reconstructed from the joint angles, which is required for the definition of the kinematic forward model (see next paragraph).

A forward model was defined, mapping joint angles onto six task-related variables: step length (*stepX*), step width (*stepY*), CoM position relative to the back foot (*bCoMX, bCoMY*), CoM position relative to the front foot (*fCoMX, fCoMY*). The measures were computed after projecting the foot (back toe, front heel) and CoM positions on the horizontal plane. Thus, the step length and width were defined as the fore-aft and lateral distance between the back toe and front heel marker at the time of heel strike. The CoM and step positions were defined in such a way that they typically have positive values and that *step* = *bCoM*+*fCoM*.

Model errors (deviations between measured and fitted task variables) were small, with an average of 3.6mm (SD: 2.22mm) in the fore-aft direction, and 5.70mm (SD: 2.25mm) in the lateral direction. No systematic differences between the age groups were found (two-way ANOVA with factors Age Group and Speed, separately for each task variable), except for step width [main effect of Age Group, F(1,30) = 5.81, p = 0.03, η^2^ = 0.13].

### Covariation Analysis

Coordination among joint angles with respect to the six task variables was assessed by a linearized version of the covariation by randomization method [10], separately for left and right heel strikes in each trial [4], [19], [20]. Briefly, this analysis assesses the effect of removing pairwise correlations between joint angles from different joints on the variability of task variables. This is done by setting corresponding entries of the covariance matrix *C* to zero (yielding the “decorrelated” covariance matrix *C_0_*) and computing task variability using the linearized forward model, i.e., its Jacobian matrix J. The empirical and decorrelated task variability (for the specific task variable defined by the forward model) are then computed as 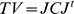 and 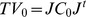, respectively. Covariation is quantified by the covariation index, 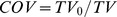.

Covariation stabilizing the task variable under consideration is present when *TV_0_>TV*, or equivalently, when *COV>1*. As *TV_0_* is the amount of task variability predicted in the absence of coordination of the DOF, the effect of motor-equivalent coordination stabilizing the task variable under consideration is reflected in reduction of *TV* relative to *TV_0_.* Hence, the stronger the motor-equivalent coordination with respect to a task variable, the greater the covariation index *COV.*

### Step-CoM correlations

CoM-related measures are likely to be prioritized over step-related measures since they are more directly related to body equilibrium. Shifting the level of analysis (from joint angles to CoM positions), we ask whether the CoM-related measures *fCoM* and *bCoM* covary in a way that stabilizes or destabilizes the step measures. Since s*tep* = *bCoM*+*fCoM* (for both the *X* and *Y* dimension), this is equivalent to the question whether *bCoM* and *fCoM* are positively or negatively correlated. Thus, step-CoM correlations were defined as
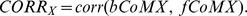


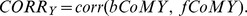



Positive correlations indicate that the corresponding step measure (*stepX* or *stepY*) is destabilized by the relation between the two CoM-related measures, while negative correlations indicate stabilization.

### Statistical Analysis

Statistical analyses were carried out in R [21], [22]. To correct for non-normal distribution, covariation indices were log-transformed [23]. The step-CoM correlations were transformed using Fisher’s *z*' transformation. After transformation, the dependent variables of each participant were averaged across heel strike events (left, right) and trials within the same speed.

Covariation indices were submitted to a repeated measures ANOVA with between-subject factor Age Group (younger, older) and within-subject factors Speed (3 levels: 80%, 100%, 120%) and Task Variable (3 levels: *step, bCoM, fCoM*), separately for the fore-aft (X) and lateral (Y) direction. Planned analyses (2-way ANOVAs) were performed, assessing the effect of Age Group separately for each Task Variable and the effect of Task Variable separately for each Age Group.

As comparison of mean and variability (SD) scores of the different task variables cannot directly compared to each other, these were analyzed separately for each task variable by two-way repeated measures ANOVAs with factors Age Group and Speed. Step-CoM correlations were submitted to a repeated measures ANOVA with factors Age Group and Speed, separately for the fore-aft (X) and lateral (Y) direction.

In the presence of interaction effects, post-hoc analyses (unpaired and paired t-tests), correcting for multiple comparisons [24], were used to assess differences between Age Groups and Task Variable, respectively.

The alpha level for statistical significance testing was set to 0.05. Effect sizes for ANOVA are reported as generalized eta-squared η^2^ [25]. Greenhouse-Geisser corrections for asphericity were applied were appropriate. All statistically significant effects (p<0.05) are reported.

## Results

### Means and Variability of Task-related Variables

**Figure 1:**
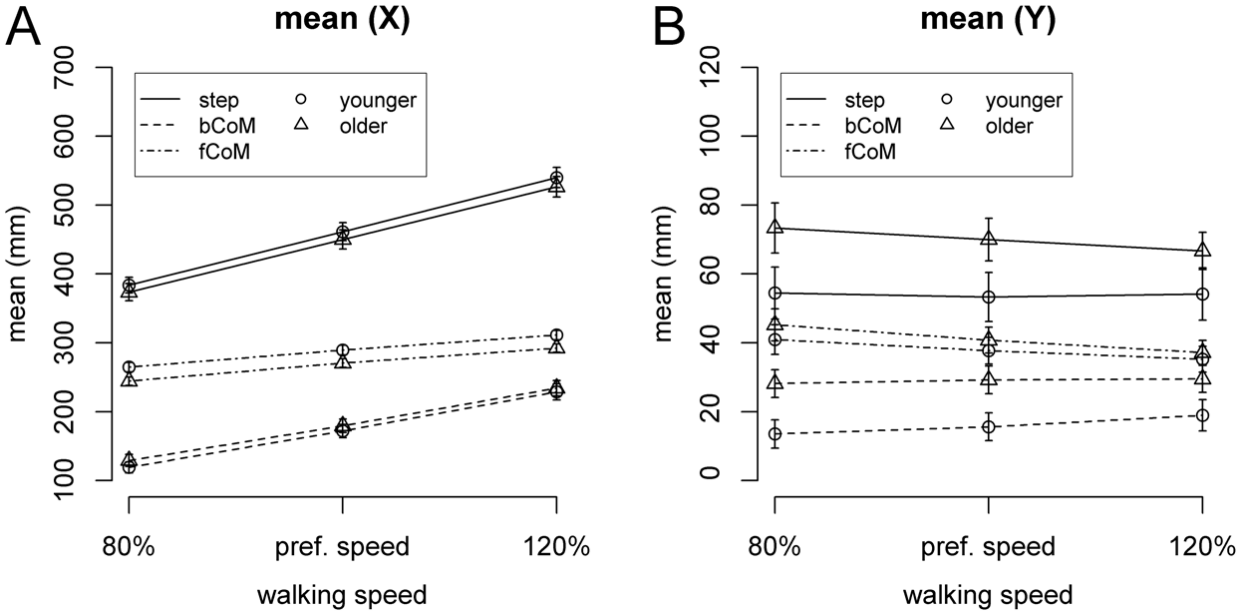
Mean of task variables in fore-aft (A) and lateral (B) direction as a function of walking speed and age group. Error bars represent SEM.

Mean values of the different task variables are plotted in Figure 1, separately for the fore-aft (Fig. 1A) and lateral (Fig. 1B) direction. In the fore-aft direction (X), main effects of Speed were found for step length [F(2,60) = 1063.4, p<0.001, η^2^ = 0.59], CoM relative to back foot [F(2,60) = 779.3, p<0.001, η^2^ = 0.56], and CoM relative to front foot [F(2,60) = 625.9, p<0.001, η^2^ = 0.37], with mean values increasing with walking speed. For CoM relative to the front foot, an additional main effect of Age Group was found [F(1,30) = 4.46, p = 0.043, η^2^ = 0.13], indicating larger values in younger adults.

In the lateral direction (Y), main effects of Speed were found for CoM relative to back foot [F(2,60) = 6.42, p = 0.003, η^2^ = 0.007] and CoM relative to front foot [F(2,60) = 31.1, p<0.001, η^2^ = 0.032]. In addition, a main effect of Age Group for CoM relative to the back foot [F(1,30) = 5.13, p = 0.03, η^2^ = 0.14] was found.

**Figure 2:**
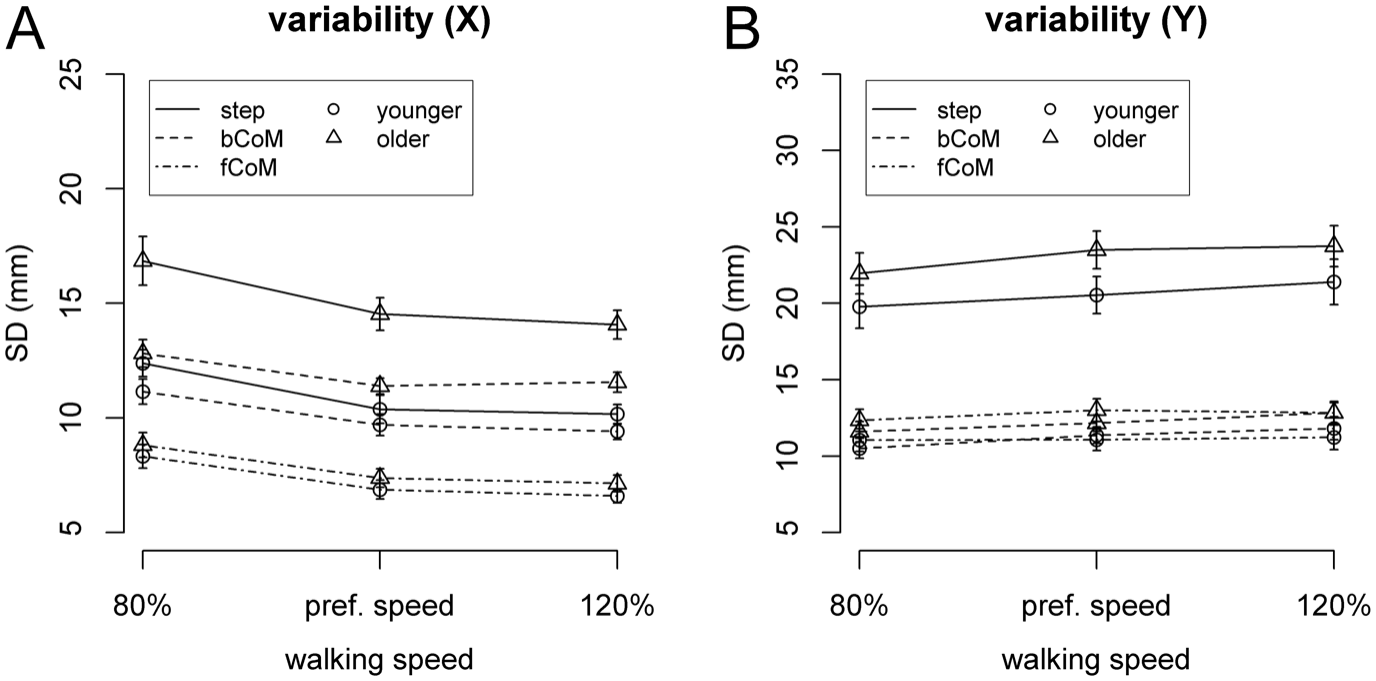
Empirical variability of task variables in fore-aft (A) and lateral (B) direction as a function of walking speed and age group. Variability is represented as standard deviation rather than variance, i.e. the square root of *TV*. Error bars represent SEM.

Variability (SD) scores of the different task variables are plotted in Figure 2. In the fore-aft direction (X), significant main effects of Speed were found for step length [F(2,60) = 12.0, p<0.001, η^2^ = 0.14], CoM relative to back foot [F(2,60) = 9.93, p<0.001, η^2^ = 0.13], and CoM relative to front foot [F(2,60) = 22.3, p<0.001, η^2^ = 0.17]. Significant main effects of Age Group were found for step length [F(1,30) = 30.9, p<0.001, η^2^ = 0.37] and CoM relative to back foot [F(1,30) = 13.7, p<0.001, η^2^ = 0.20], with higher variability in older compared to younger adults.

In the lateral dimension, variability scores showed main effects of Speed for step width [F(2,60) = 3.28, p = 0.04, η^2^ = 0.02] and CoM relative to back foot [F(2,60) = 6.55, p = 0.003, η^2^ = 0.042].

### Age-general Pattern of Motor-equivalent Coordination and Differences between Task Variables

**Figure 3:**
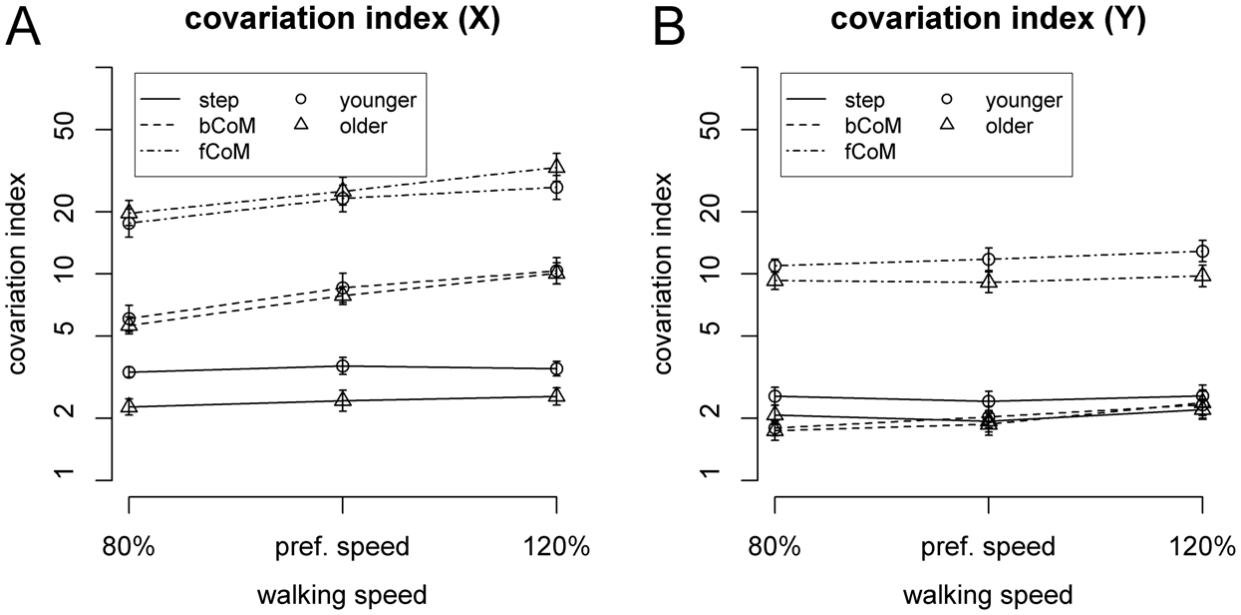
Covariation index in the fore-aft (A) and lateral (B) direction as a function of walking speed and age group. Error bars represent SEM.

Covariation indices for the different task variables are plotted in Figure 3 on a logarithmic scale. One-sample t-Tests confirmed that all task variables were stabilized by motor-equivalent coordination (log-transformed covariation indices greater than zero), for both age groups and at all walking speeds (p<0.001 in all cases, Bonferroni-Holm corrected).

In the fore-aft direction (Figure 3A), separate ANOVAs (Speed × Task Variable) for younger and older adults showed similar patterns of results in both age groups, with main effects of Speed [younger adults: F(2,30) = 26.3, p<0.001, η^2^ = 0.068, older adults: F(2,30) = 17.7, p<0.001, η^2^ = 0.11], Task Variable [younger adults: F(2,30) = 128.14, p<0.001, η^2^ = 0.69, older adults: F(2,30) = 333.1, p<0.001, η^2^ = 0.81], and a two-way interaction [younger adults: F(4,60) = 8.04, p<0.001, η^2^ = 0.028, older adults: F(4,60) = 8.51, p<0.001, η^2^ = 0.031]. Pairwise comparisons at each Speed level showed that, in both age groups and at all speeds, covariation indices were highest for the CoM relative to the front foot, followed by the CoM relative to the back foot, and lowest for step length (p<0.002 in all cases, Bonferroni-Holm corrected).

For the lateral direction (Figure 3B), separate ANOVAs (Speed × Task Variable) for younger and older adults showed similar patterns of results, with main effects of Task Variable [younger adults: F(2,30) = 141.4, p<0.001, η^2^ = 0.76, older adults: F(2,30) = 160.5, p<0.01, η^2^ = 0.72], and a two-way interaction of Speed by Task Variable [younger adults: F(4,60) = 2.85, p = 0.031, η^2^ = 0.01, older adults: F(4,60) = 4.46, p = 0.003, η^2^ = 0.01]. Pairwise comparisons between task variables at each Speed level showed that, in both age groups and at all speeds, covariation indices were higher for the CoM relative to the front foot compared to both CoM relative to the back foot and step width (p<0.001 in all cases, Bonferroni-Holm corrected).

Thus, the general pattern of motor-equivalent stabilization was similar across age groups, with all task variables under consideration showing significant covariation, and with higher covariation indices for the CoM relative to the front foot, compared to all other task variables.

### Age differences in Motor-equivalent Stabilization of Task Variables

The main research question of the present study concerned age differences in the pattern of motor-equivalent stabilization of different task variables (see Fig. 3). In the fore-aft direction (Figure 3A), the omnibus ANOVA revealed main effects of Speed [F(2,60) = 40.11, p<0.001, η^2^ = 0.09], Task Variable [F(2,60) = 407.0, p<0.001, η^2^ = 0.76], and two-way interactions of Age Group by Task Variable [F(2,60) = 5.84, p = 0.006, η^2^ = 0.04], and Speed by Task Variable [F(4,120) = 16.4, p<0.001, η^2^ = 0.029]. ANOVAs assessing effects of Age Group and Speed separately for each Task Variable, showed a main effect of Age Group for step length [F(1,30) = 11.6, p = 0.002, η^2^ = 0.21], with lower covariation indices in older adults. Main effects of Speed were found for CoM relative to back foot [F(1,30) = 75.28, p<0.001, η^2^ = 0.17] and CoM relative to front foot [F(1,30) = 37.1, p<0.001, η^2^ = 0.10].

In the lateral direction (Figure 3B), the omnibus ANOVA revealed main effects of Speed [F(2,60) = 5.14, p = 0.012, η^2^ = 0.019], Task Variable [F(2,60) = 298.1, p<0.001, η^2^ = 0.74], and a Speed by Task Variable interaction [F(4,120) = 6.28, p = 0.002, η^2^ = 0.01]. Subsequent ANOVAs assessing effects of Age Group and Speed separately for each Task Variable showed a main effect of walking speed for CoM relative to the back foot [F(2,60) = 20.0, p<0.001, η^2^ = 0.06]. In particular, no systematic effects of Age Groups were found for any of the task variables in the lateral direction.

Summing up, older participants showed lower covariation indices with respect to step length, indicating weaker motor-equivalent stabilization of this task variable across walking speeds. In contrast, no reliable differences between the age groups were observed for any of the other task variables.

### Relationship between CoM and Step Fluctuations

**Figure 4:**
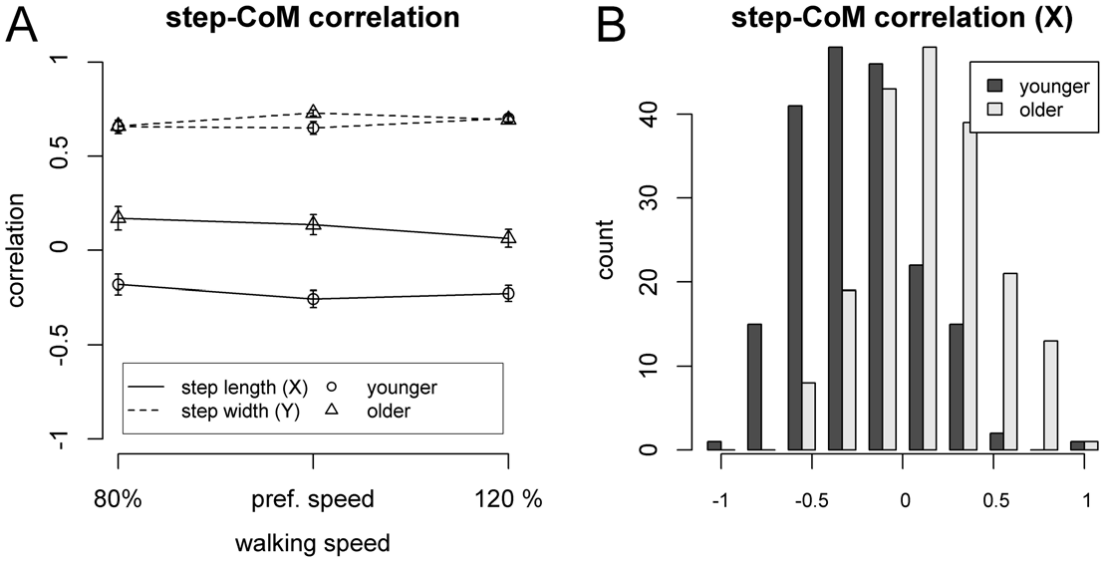
Step-CoM correlations. A. Correlation coefficient, as a function of direction (step length, step width), walking speed, and age group. Error bars represent SEM. **B.** Histogram of correlation coefficients in the fore-aft (X) direction.

Figure 4A shows step-CoM correlations, as a function of Age Group, Speed and Direction (fore-aft, lateral). One-sample t-tests (separately for each Age Group and Speed) showed that step-CoM correlations with respect to step width were significantly larger than 0 in both age groups [all t(15)>18, p<0.001, corrected]. With respect to step length, step-CoM correlations were negative at all speeds in young men [t(15)<-3, p<0.05], and tended to be positive in older men [80% pref. speed: t(15) = 2.71, p = 0.048; pref. speed: t(15) = 2.55, p = 0.048].

The ANOVA assessing the effect of Age Group and Speed on step-CoM correlations for step width showed an Age Group by Speed interaction [F(2,60) = 3.85, p = 0.033, η^2^ = 0.036]. Post-hoc analyses of age-effects did not show any reliable Age Group difference (p>0.2 at all Speeds). In contrast, the corresponding analysis for step length (X) showed a main effect of Age Group [F(1,30) = 36.5, p<0.001, η^2^ = 0.43]. Step-CoM correlations with respect to step length were larger (less negative) in older compared to younger men (see histogram in Fig. 4B).

Thus, step-CoM correlations in the lateral dimension were similar between young and older men, and consistent with the findings of a previous study in young men (Verrel et al. 2011). In contrast, older and younger participants differed in the fore-aft direction, with older adults showing weaker and non-significant step-length stabilization at the level of step-CoM correlations.

## Discussion

We studied adult age differences in motor-equivalent stabilization of step parameters and center of mass (CoM) position during treadmill walking in healthy younger and older men. Stabilization of task-related variables was assessed by means of a covariation index, comparing variability in actual and covariation-free (surrogate) data. In addition, we computed step-CoM correlation indices to quantify the extent to which the positions of CoM relative to the back and front foot are coordinated in a way that stabilizes or destabilizes the step parameters.

In agreement with a previous study with young men [4], participants in both age groups showed reliable covariation for each of the task variables under consideration, indicating that their gait was stabilized by motor-equivalent coordination of the major joints of their bodies. Across participants from both age groups and across a range of walking speeds, covariation was strongest for the CoM position relative to the front foot, which is arguably the task variable most directly related to maintenance of body equilibrium.

On top of the overall similarity in covariation patterns between the two age groups, older men showed weaker covariation with respect to step length than younger men. This finding was complemented by age differences in step-CoM correlations in the fore-aft dimension (step length), which also were weaker (and not significantly different from zero) in older men. In contrast, no consistent age differences were present regarding stabilization of the CoM position in the fore-aft direction, or regarding stabilization of any of the task variables in the lateral direction.

Taken together, these findings suggest an adult developmental pattern in which stabilization of step length, a variable that is of lower functional priority (e.g., in relation to the spatiotemporal regularity of the gait pattern) is reduced in old age, whereas stabilization of variables that are more critical for maintaining body equilibrium (e.g., CoM position) is preserved.

### Motor-equivalent Stabilization of Task-related Variables

According to our results, both young and older adults showed motor-equivalent coordination stabilizing the task-related variables under consideration. More specifically, across age groups, joint angle variability across the body was organized in a way minimizing variability at the “ouput level” (e.g., step length or CoM position). This finding extends a previous study with only young adults [4] to adults above the age of 65 years. In addition, as in that previous study, both younger and older adults showed higher covariation indices (indicating stronger motor-equivalent stabilization) of the CoM relative to the front foot compare to all other task-related variables. Thus, priorities among these parameters appear to be preserved in healthy aging.

It should be noted that stabilization of task-related variables by motor-equivalent coordination could not have been analyzed based on variability scores of these variables alone. Variability scores can usually not be compared between task-related variables, even if they are represented in the same units, and they are difficult to interpret as absolute values. Most importantly, raw variability scores are not informative about the underlying organization of variability across the motor system, which is an important aspect of motor coordination [2], [3], [9].

### Age-related differences in Motor-equivalent Coordination

Age-related reductions in the use of motor equivalence have previously been observed experimentally in multi-finger force control [5], [6] and manual pointing [7]. Given that motor-equivalent stabilization of step length was weaker in older participants than in younger participants, the results of the present study support the view that multi-DOF coordination declines with advancing adult age. In addition, the present results also suggest that the older adults’ motor system may deal with this decline in a selective and adaptive manner, sparing coordination with respect to the functionally most relevant task variable, that is CoM position relative to the front foot, thereby ensuring maintenance of whole-body equilibrium.

In contrast to a previous study in manual pointing [7], in which weaker motor-equivalent coordination in older adults did not result in poorer performance, the present study did find higher variability at the ``output level'' for one of the task-related variables under consideration (step length). This indicates that age-related differences in motor-equivalent coordination may be reflected both at the level of joint angles (reduction in variability, as in the pointing study) and at the task level (increase in variability, as in the present study for step length). The different pattern of results observed in the two studies may be due to differences in task dynamics and task complexity: compared to walking, manual pointing is a biomechanically simpler task with a unidimensional “goal structure” (a single task variable, namely the fingertip position). Therefore, it may be more amenable to the strategy of reducing overall joint variability, which might not be available for walking, which requires concurrent stabilization of multiple task variables and which has stronger intrinsic dynamics and stability constraints.

Age-related changes in gait stability have previously been studied using perturbation paradigms. Responding to balance perturbations depends on appropriate whole-body coordination, including the exploitation of motor-equivalence to stabilize the CoM [26], suggesting a link between such perturbation measures and motor-equivalent stabilization of gait patterns during unperturbed walking. However, while older adults have been found to perform less efficiently in perturbation paradigms [27], our present study did not find age differences in motor-equivalent CoM stabilization during unperturbed walking. Thus, responding to external perturbation may involve different or additional control processes compared to those governing the stabilization of unperturbed movement, and these may be differentially affected by adult aging.

Gait stability has also been analyzed using nonlinear dynamic measures, assessing how small, naturally occurring fluctuations are increased or attenuated over time [28]–[30]. Dynamic measures analyze the temporal evolution, while covariation measures indicate how fluctuations in different DOF across the body compensate for each other to stabilize certain task variables. Nonlinear analyses have found evidence for age-related decline in dynamic stability [31], [32]. The relationship between these the two notions of stability – dynamic versus motor-equivalent – and associated age-related differences in each of them are important targets for future research.

### Methodological Considerations

The number of steps (20) used to assess gait variability presents a limitation of the present study with respect to determining accurate individual variability scores [33]. However, we are confident that the main findings with respect to the covariation indices, that is the age group difference and the contrast between step and CoM stabilization, are not undermined by this limitation: the present results were found consistently across a range of walking speeds, after prolonged continuous walking on the treadmill (ruling out age differences in adaptation), and with a similar number of movement repetitions (step postures) as used in previous studies, for instance on manual pointing [7], [34].

Younger and older participants chose similar preferred speeds for walking on the treadmill. However, spontaneous overground walking was significantly faster in younger participants than in older participants, indicating that the older participants might have walked closer to their individual “speed limits” during the experiment. Importantly, this difference cannot explain the observed age difference in step length variability (because step length variability decreased with increasing walking speed), nor can it explain the absence of an age effect in step width variability (which tended to increase with walking speed).

Regarding generalizability, we took several measures to minimize potential differences between treadmill and overground walking. First, the range of walking speeds was defined relative to self-selected “preferred” walking speeds. Second, the treadmill was embedded into the ground, so that participants walked at the level of the surrounding floor. Third, a virtual environment consisting of a straight path was projected on a screen to provide a more naturalistic context for walking [35]. Hence, we are confident that the age differences in the motor-equivalent stabilization of gait patterns observed in the present study are likely to generalize to overground walking.

It may appear surprising that the participants of the present study showed evidence for motor-equivalent stabilization of step length, while previous studies have shown that step length covaries with step time in a way that stabilizes walking speed [16], [36]. These two findings are, however not contradictory. First, it is possible to simultaneously stabilize multiple performance variables (as shown in the present study, for step parameters and CoM position). Second, the abovementioned studies differ from the present study in the duration of the walking trial, and in the number of steps analyzed (20 in the present study, at least 256 in the above studies). Over these longer time periods, persistence of fluctuations in step length is likely to be more pronounced. Finally, the studies differ in the level of analysis. We analyzed stabilization of step length, both in terms of covariation across joints of the entire body and in terms of correlation of the CoM position relative to front and back foot. In contrast, the abovementioned studies analyzed stabilization of walking speed in terms of the correlation between step duration and step length.

Finally, body postures were represented in terms of joint angles in the present study, as done in most studies on motor-equivalent coordination we are aware of. It has been shown that measures of motor-equivalent coordination are affected by the choice of coordinate system [37]. The representation in terms of joint angle was chosen because it has the advantage (compared to, e.g., elevation angles) that the DOF are independent. By definition, joint angles can be freely combined without violation of anatomical constraints of different joints. In contrast, independent permutation of the orientation of neighboring segments (i.e., elevation angles), as done in randomization methods, may result in anatomically impossible joint configurations.

### Conclusion

Relative to young participants, healthy older participants display reduced coordination stabilizing step length during treadmill walking. In contrast, motor-equivalent coordination was spared from age-related decline with respect to step width and center of mass position relative to the front and back foot. Older adults may deal with coordination impairments in an adaptive fashion, prioritizing variables with respect to maintenance of whole-body equilibrium.
