# Correction: Older Adults Show Preserved Equilibrium but Impaired Step Length Control in Motor-Equivalent Stabilization of Gait

**DOI:** 10.1371/annotation/3d535446-2379-4ade-8db9-ae6547e9b875

**Published:** 2013-08-08

**Authors:** Julius Verrel, Martin Lövdén, Ulman Lindenberger

Reference number 36 refers to the incorrect work. The correct reference is the following:

Dingwell JB, John J, Cusumano JP (2010) Do Humans Optimally Exploit Redundancy to Control Step Variability in Walking? PLoS Comput Biol 6(7): e1000856. doi:10.1371/journal.pcbi.1000856 

